# Design and Evaluation of a Potential Non-Invasive Neurostimulation Strategy for Treating Persistent Anosmia in Post-COVID-19 Patients

**DOI:** 10.3390/s23135880

**Published:** 2023-06-25

**Authors:** Desirée I. Gracia, Mario Ortiz, Tatiana Candela, Eduardo Iáñez, Rosa M. Sánchez, Carmina Díaz, José M. Azorín

**Affiliations:** 1 Brain-Machine Interface Systems Lab, Miguel Hernandez University of Elche, 03202 Elche, Spaineianez@umh.es (E.I.); jm.azorin@umh.es (J.M.A.); 2 Engineering Research Institute of Elche—I3E, Miguel Hernandez University of Elche, 03202 Elche, Spain; 3 Department of Neurology, Hospital General Universitario Dr. Balmis de Alicante, 03010 Alicante, Spain; 4 Valencian Community Foundation for the Management of the Institute of Health and Biomedical Research of Alicante (ISABIAL), 03010 Alicante, Spain; 5 Valencian Graduated School and Research Network of Artificial Intelligence—ValGRAI, 46022 Valencia, Spain

**Keywords:** anosmia, COVID-19, neurostimulation, electroencephalography

## Abstract

A new pandemic was declared at the end of 2019 because of coronavirus disease 2019 (COVID-19). One of the effects of COVID-19 infection is anosmia (i.e., a loss of smell). Unfortunately, this olfactory dysfunction is persistent in around 5% of the world’s population, and there is not an effective treatment for it yet. The aim of this paper is to describe a potential non-invasive neurostimulation strategy for treating persistent anosmia in post-COVID-19 patients. In order to design the neurostimulation strategy, 25 subjects who experienced anosmia due to COVID-19 infection underwent an olfactory assessment while their electroencephalographic (EEG) signals were recorded. These signals were used to investigate the activation of brain regions during the olfactory process and identify which regions would be suitable for neurostimulation. Afterwards, 15 subjects participated in the evaluation of the neurostimulation strategy, which was based on applying transcranial direct current stimulation (tDCS) in selected brain regions related to olfactory function. The results showed that subjects with lower scores in the olfactory assessment obtained greater improvement than the other subjects. Thus, tDCS could be a promising option for people who have not fully regained their sense of smell following COVID-19 infection.

## 1. Introduction

At the end of 2019, a new pandemic was declared due to the worldwide major health problem caused by coronavirus disease 2019 (COVID-19), which was produced by a new type of coronavirus (SARS-CoV-2). Initially, COVID-19 was considered a respiratory disease associated with pneumonia symptoms. However, neurological conditions caused by the disease were described in several studies [1,2]. Different patterns have been detected in the electroencephalographic (EEG) signals of COVID-19 patients with neurological symptoms caused by the disease [1]. Some of these patterns were found in the frontal areas of the brain. This fact reinforced the theory that SARS-CoV-2 enters the central nervous system (CNS) by the olfactory structures and spreads through the frontal lobes [2]. This hypothesis concurs with the significant percentage of patients suffering anosmia, i.e., loss of smell, at the initial stages of the infection [3]. Indeed, loss of smell is a common COVID-19 symptom, varying its incidence from 53% to 17% depending on the variant causing the infection [4]. This means that there are around fifteen million people suffering from this condition around the world [5].

The causes of this olfactory dysfunction are yet unknown. It has been proven that there is not a direct relationship with disease severity or viral load [6,7]. Some theories propose that it is caused by direct cell injury or secondary inflammation from viral infection of the olfactory pathway [8,9,10,11]. The theory of the olfactory dysfunction being caused by the effects of the infection on the nasal mucosa fails to explain the persistent anosmia [6,12,13,14] since cell damage in the nasal mucosa is largely repairable.

Regardless, in most cases, the symptoms spontaneously resolve within weeks. Indeed, around 75% of patients recover their sense of smell in less than a month. However, between 5% [5] and 7.5% [15] of patients do not recover their sense of smell after half a year or even two years.

Even though the loss of smell is not a new disease (approximately 15% of the Spanish population suffer this disease [16]; this number is 5% when taking into consideration the world population [17]), there is still no cure for it. Although some treatments can be prescribed, including olfactory training or drugs such as corticosteroids [18,19], they lack specificity and efficacy [17]. In order to propose a new procedure to allow patients to recover their normal sense of smell, it is necessary to gain more knowledge about the physiological anomalies that occur due to COVID-19 infection. This would also be helpful as it would provide a better understanding of the areas affected by other causes of loss of smell, such as neurodegenerative diseases. Anosmia is mainly caused by head trauma, viral infections, rhinosinusitis, nasal polyposis, and neurodegenerative diseases, such as Parkinson’s or Alzheimer’s disease [20]. It has been proven that COVID-19 infections do not provoke a rupture of the olfactory nerve and thus cause inflammation as in the case of rhinosinusitis; instead, it has been found that COVID-19 alters olfactory structures, which are also damaged in the neurodegenerative anosmic cases [21,22].

Since it has been demonstrated that electrical stimulation in the nose induces a sense of smell [23], there have been attempts to create an “electric nose” as an equivalent of a cochlear implant, which is used to recover the sense of hearing [24,25,26]. However, those initial studies used a very invasive kind of stimulation that, in some cases, required an ethmoidectomy [27]. Thus, the current approaches tend to non-surgical procedures.

Due to our greater knowledge about certain brain regions, brain stimulation therapies have become important alternative options. Transcranial direct current stimulation (tDCS) seems to have the most promise due to its cost, simplicity of application, and favorable tolerability profile [28]. tDCS has been shown to have positive benefits in the treatment of neurological and psychiatric conditions, as well as in the recovery of cognitive, motor, and sensory abilities [29,30]. Researchers are also investigating the efficiency of using tDCS to treat different symptoms caused by COVID-19 infection, such as dyspnea [31], pain, fatigue [32], and stress [33].

This paper presents a new potential non-invasive neurostimulation strategy based on tDCS with the aim of treating persistent anosmia in post-COVID-19 patients. In order to investigate the activation of brain regions during the olfactory process and identify which regions would be suitable for tDCS electrode placement, 25 subjects underwent an olfactory assessment while their EEG signals were recorded. Afterwards, 15 subjects participated in the evaluation of the tDCS strategy.

## 2. Materials and Methods

This section explicates the attributes of the patients who participated in the study, along with the materials employed in the experimental procedures involving concurrent EEG registration and olfactory assessments and the application of the neurostimulation strategy. Furthermore, we expound upon the analysis conducted on the various acquired data.

### 2.1. Participants

The recruitment process of participants was significantly influenced by various factors. A significant proportion, more than 90% [5,15], of patients who experienced loss of smell as a symptom of COVID-19 infection recover their olfactory function without requiring any treatment. To minimize the potential influence of unexamined variables, it was necessary to ensure that the patients had surpassed the period of potential spontaneous recovery and did not have any other brain-related conditions. Another notable limitation was the scheduling of sessions, as they needed to be convenient in terms of time and location for participants to travel to the hospital, considering that the study was part of a year-long project.

Eventually, 25 patients were recruited from July 2021 to February 2022 to participate in the study: 12 females/13 males, aged 19 to 55 years. Their time since COVID infection ranged from 12 to 24 months. These patients were recruited considering the following inclusion and exclusion criteria.

- Inclusion criteria: (1) patients who suffered loss of smell after infection by SARS-COV-2 (confirmed through positive PCR); (2) age between 18 and 55 years old; and (3) normal examination without clinic or exploratory data that hint at neurodegenerative diseases.
- Exclusion criteria: (1) fluctuating or paroxysmal loss of smell; (2) smoking; (3) use of drugs that could affect the sense of smell; (4) history in first-degree relatives of neurodegenerative diseases; (5) diseases that could affect the sense of smell; (6) pregnancy; and (7) use of an artificial pacemaker.

Afterwards, only 15 subjects participated in the evaluation of the neurostimulation strategy.

The Ethics Committee for Drug Research of the Department of Health of Alicante—General Hospital approved the present study (Ref. CEIm: PI2022-035 and Ref. ISABIAL: 2022-0035). The patients were informed about the experimental procedure, after which they signed written informed consent forms. The study was conducted according to the World Medical Association’s Declaration of Helsinki.

### 2.2. Devices

**Figure 1:**
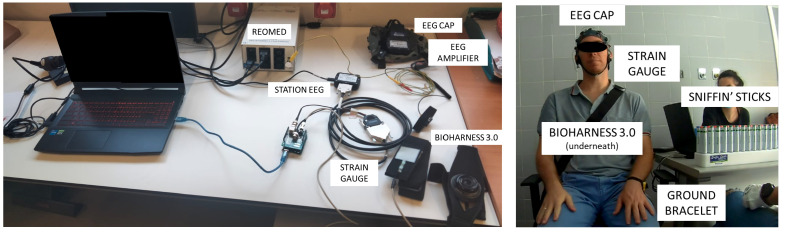
Material employed in the performance of the olfactory assessments.

Figure 1 shows the material employed to carry out the experimental procedure.

The Sniffin’ Sticks test (Burghardt, Germany) was used as olfactory stimuli [34]. The version employed was the 2-phenylethanol extended smell test, which consisted of three tests:

1. A threshold test conducted with 48 Sniffin’ Sticks (32 blanks and 16 dilutions of 2-phenylethanol with different concentrations. Stick number 16 was the one with the lowest fragrance concentration, and stick number 1 had the highest concentration).
2. A discrimination test conducted with 48 Sniffin’ Sticks (16 triplets, in which each one has two sticks with the same odor, while the other one is a different scent and is thus the target).
3. An identification test conducted with 16 Sniffin’ Sticks (with different everyday smells).

EEG data were recorded using the g.Nautilus PRO FLEXIBLE (g.tec medical engineering GmbH, Austria) with a wet system of 32 non-invasive electrodes. The 32 electrodes selected for acquisition were positioned following the 10–10 distribution of the international system at FP1, FP2, AF3, AF4, F7, F3, FZ, F4, F8, FC5, FC1, FC2, FC6, T7, C3, CZ, C4, T8, CP5, CP1, CP2, CP6, P7, P3, PZ, P4, P8, PO7, PO3, PO4, PO8, and OZ. Another two electrodes were used as ground and reference electrodes, which were positioned in the right ear lobe and the AFZ international system position, respectively. The data were recorded with a sampling rate of 500 Hz via a Wi-Fi connection through the g.NEEDaccess software (g.tec medical engineering GmbH, Schiedlberg, Austria), and the data were forwarded to the MATLAB (version 2022a) platform, where the data were stored, processed, and analyzed.

The respiratory signal was recorded to label inhalation events during the EEG register. The respiratory signal was recorded using a BioHarness 3.0 (Zephyr Technology Corporation, Annapolis, MD, USA) with a sampling rate of 18 Hz. Data were acquired via a Bluetooth connection through the Bluetooth BioHarness Test Application (Zephyr Technology Corporation, Annapolis, MD, USA).

A strain gauge was used to mark the instant where the subject could start breathing in. This strain gauge was pressed by the experimenter’s assistant. The gauge was placed on the shoulder of the participant, attached to a belt, and connected to an Arduino UNO Rev 3 board (Arduino, Boston, MA, USA) which was wired to the EEG station. The threshold needed to activate the gauge by pressing it was specified via a custom code on the Arduino IDE platform (Arduino, Boston, MA, USA).

REOMED I 600 (REO AG, Solingen, Germany) was used to ensure the safety of patients, reducing the leakage current of the electrical systems. Furthermore, the patients were electrically grounded with the use of a bracelet.

The tDCS strategy was applied with a Starstim 8 device (Neuroelectrics, Barcelona, Spain) with a wet system of 8 non-invasive electrodes. The electrodes employed were spherical with a diameter of 1 cm. The 5 electrodes selected for stimulation were positioned following the 10–10 distribution of the international system at FP1, FP2, O1, O2, and IZ. Two extra electrodes were used as ground and reference electrodes, which were both positioned in the right ear lobe. The configurations were designed with the NIC2.1.15 software (Neuroelectrics, Barcelona, Spain).

### 2.3. Procedure

The experiment was divided into three stages: an initial olfactory test, five sessions of stimulation, and a final olfactory test.

#### 2.3.1. Initial Olfactory Test

The olfactory test was divided into eight phases, which are described below.

In phases 1, 4, 6 and 8, the subject remained relaxed for two minutes with their eyes closed. These phases provided information about the basal state of the EEG data. They were also relevant as break time from the different olfactory tests in order to avoid olfactory saturation.

In the second phase, an initial test of the threshold test took place, showing the subject the triplet of sticks with the higher concentration. The target stick was presented for 5 s. After 30 s, to allow for olfactory adaptation, the other two blank sticks were presented for 5 s each with a 5 s interval time between them, which was necessary to assure that there was not saturation. This step allowed the subject to recognize the target odor during the following trials.

The threshold test was performed in the third phase. This test allows the minimum concentration that the subject was able to smell to be determined. The trials were performed following a staircase procedure, i.e., the concentration of the triplets was increased or decreased after each trial. They were increased or decreased one level at a time, except in the first set, where the increase was on the order of two levels. The test was divided into seven sets of turning points, with the direction of the staircase changing after ending a set, which happened when a turning point was reached. In the sets with an increasing direction (odd numbers), the concentration was increased when the subject was not able to correctly identify which stick had the odor, and a turning point happened if the stick was properly identified twice in a row. In the sets with a decreasing direction (even numbers), the concentration was decreased after each proper identification (again, it was necessary for these to be correctly identified twice), and the turning point was defined when a misidentification happened. The first set started with the stick with the lower concentration, and the following sets started with the stick that was one step lower if the previous set was an odd number or higher if it was an even number. The number of events recorded was not prefixed as the length of each set was defined according to the correct answers of the participant. The time sequence was similar to that used in the third phase, but all the intervals between sticks were of approximately five seconds, keeping a minimum of 30 s between triplets to allow the subject to provide an answer about the stick perceived as the correct one. The presentation of the stick triplets was randomized in a fixed way (the same randomization was used for all the patients). The score of this test was calculated as the average of the stick number for the last four breaking points.

In the fifth phase, the discrimination test was performed. The subject had to identify which stick had a different odor in the triplet. The sequence used was the same as in the third phase. The score of this test was calculated as the number of correct answers.

The identification test was performed in the seventh phase. In this test, the subject had to recognize sixteen everyday smells, wih one stick per each trial. Each Sniffin’ Stick was presented for 5 s, and then a 30 s time interval was set between trials, in which the subject identified the odor smelled by answering a four-choice question. The score of this test was calculated as the number of correct answers.

Since some of the Sniffin’ Sticks are visually distinguishable, the first two tests require the subject to have their eyes closed. In order to obtain the same level of visual feedback during the EEG recording, the subjects were required to close their eyes during all tests to acquire the EEG signals under the same conditions.

To avoid any auditory evoked potentials, the instruction provided to the subject to start the action of breathing in was not oral; it was instead provided by touching them on the shoulder, which is a zone that has a lower representation in the somatosensory cortex.

Even though this test allows the performance of each nostril to be evaluated individually and jointly, only the last option was conducted in order to avoid overly long tests. While presenting the pen, it was placed at a distance of 2 cm from the nostrils, being careful to ensure that it never touched the nose or the hands of either the operator or the subject.

After each trial, even if the subject was unsure, they were forced to give an answer. The cooperation of the person was very important since this is a semi-objective test and random answers will highly influence the reliability of the results of the test [35].

#### 2.3.2. Stimulation Sessions

**Figure 2:**
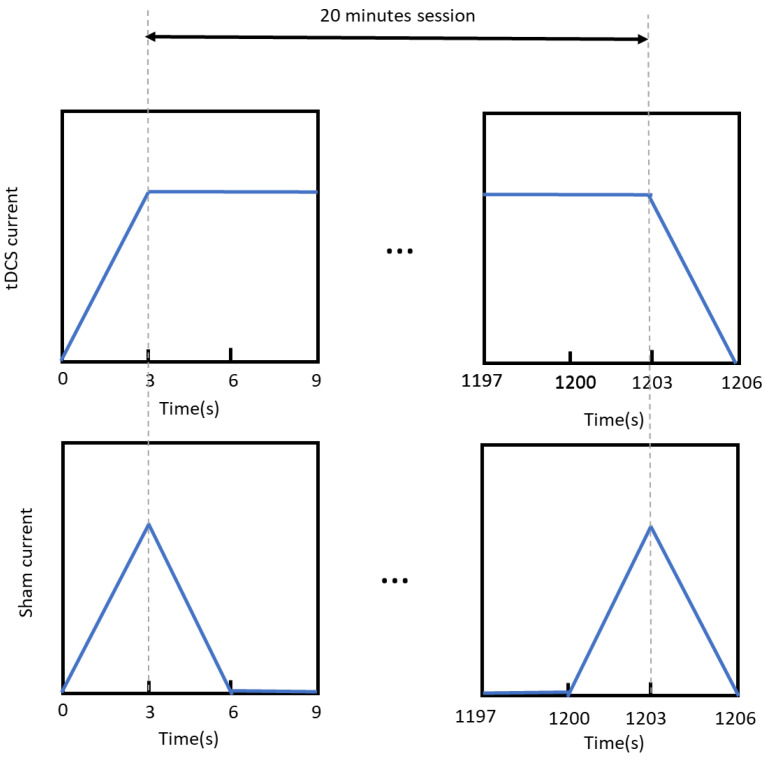
Comparison of the application of the anode current between the different groups of stimulation. In the active stimulation group, the current was constant throughout the whole procedure, while in the sham stimulation group, the current was only applied during a ramp-up of 3 s and a ramp-down of 3 s at the beginning and the end of the procedure.

Patients were initially divided into two groups according to their personal testimony of having recovered their sense of smell. This division was corrected after performing the first olfactory test. Thus, only people who obtained a score lower than expected for half of their age group were allocated to perform the stimulation session. The patients for the stimulation sessions were randomly divided, with stratification based on gender, into two groups. One received active stimulation, while the other received a sham treatment. This division allowed to study the placebo effect of the experiment. In both cases, each session lasted 20 min plus an initial ramp-up of 3 s and a ramp-down at the end of another 3 s. In the active group, the current was constant through the whole session, while in the sham, the current was turned down after the initial ramp-up and turned on before the final ramp-down; each change had a duration of 3 s. In both procedures (see Figure 2), the subjects were not capable of identifying the group to which they belonged. The stimulation was performed in five sessions on different days within a maximum of two weeks.

In order to design the electrode configuration for the tDCS strategy, we took into consideration the information available from the state of art, as well as the EEG data registered during the first olfactory assessment.

#### 2.3.3. Final Olfactory Test

Less than two weeks after the last stimulation session, the olfactory test was repeated, following the same process as the initial test with only the following difference. In the discrimination and identification initial tests, the sticks were presented following an increasing direction of the number of the stick. However, in the final tests, they were presented following a randomized order, trying to avoid a situation where the patients answered based on their memories of the first experience.

### 2.4. Data Analysis

#### 2.4.1. Initial Olfactory Assessment

The condition of the subject was assessed by adding up the scores of the three tests performed in the first olfactory test. The TDI score (threshold, discrimination, and identification) sorted those scores higher than 30.75 as normosmia, between 30.5 and 16.25 as hyposmia, and lower than 16 as a functional anosmia. The score of the subject could also be classified into a percentile performance according to their age group [36].

#### 2.4.2. Final Olfactory Assessment

The final olfactory test allowed us to analyze the evolution of the patients’ olfactory abilities, taking into consideration individual variables, such as their age, gender, kind of stimulation received, days passed between the olfactory tests, and time between the last stimulation received and the final olfactory test.

#### 2.4.3. EEG Data

All the analyses were performed offline, although the first step of the pre-processing techniques was performed during the data acquisition step. A notch filter at 50 Hz was applied as a hardware filter in order to mitigate the noise caused by the power line. The following steps were performed through the MATLAB interface, where the data were received and stored.

Firstly, the data were frequentially and spatially filtered using a second-order Butterworth state-variable band-pass filter with cut-off frequencies of 0.1 and 100 Hz to remove the DC offset and a Laplacian spatial filter to enhance the local activity. Additionally, the signal was normalized based on the data registered for the basal state using the average of the signal extreme values of the four trials with eyes closed.

The respiratory signal was employed in order to define the interval of time of interest. The first step was to resample the breathing signal to the EEG signal’s sample frequency. Then, a four-second time window was defined for each stick, considering its beginning as the first instant after the trigger mark where the participant had inhaled for at least half a second.

The olfactory response has been proven to have a fast processing speed [37,38,39,40,41]; it is thus necessary to include higher frequencies in its analysis to detect the changes provoked. However, these higher components also include high-frequency noise, which is usually removed through the averaging of numerous trials. Since in the Sniffin’ Test each stick was mostly presented once and the different sticks might present different responses due to the use of different odors and concentrations, this noise filtering through averaging was not plausible. Hence, our attention was focused on lower-frequency bands, as in [42,43,44].

Since there was not a consensus in previous studies about the frequency bands selected for analysis, a spectral study of the signals was performed. Taking into consideration that the values of latency of the olfactory evoked responses were estimated around 10 ms and 800 ms, the time window analyzed was reduced to the first second. The spectral analysis revealed that the alpha band was the one with a higher peak value during the first second after stimuli. Therefore, the EEG signals were filtered again with a second-order Butterworth state-variable band-pass filter with cut-off frequencies of 8 and 14 Hz.

#### 2.4.4. Stimulation

To determine the model that yielded the most substantial stimulation in the areas of interest, an analysis of different electrode configurations was conducted using SimNIBS simulation [45]. This analysis aimed to identify the configuration that provided the highest level of stimulation in the targeted areas.

The employed electrodes were spherical in shape, measuring 10 mm in diameter. This size is notably smaller than the conventional sponge electrodes employed in studies involving tDCS strategies, which typically range from 5 to 35 cm^2^ in area. The reduced area of the electrodes enables the more precise targeting of stimulation. In all cases, the anode electrodes delivered a current of 0.5 mA, a value limited by the electrode area utilized.

For each model, the normal component of the electric field (normE) was calculated using MATLAB for three structures. The values within a 10 mm radius were averaged for each representative coordinate following the procedure outlined in [46]. The structures related to the olfactory system studied are as follows: (1) the olfactory bulb [3.24373; 49.9385; 15.2796]; (2) the olfactory tract [5.8274; 14.4474; 14.2906]; and (3) the piriform cortex [−23.6487; −6.17281; −2.86413]. The maximum value of this variable was also retrieved to ensure that safety limits were not exceeded [47].

## 3. Results

### 3.1. Initial Olfactory Assessment

Table 1 presents the scores obtained for each individual test and for the combined test, as well as the classification corresponding to the patients’ TDI scores and the percentile they are located in.

The first codes, seen in the column labeled Subject, were reserved for the subjects who indicated a recovery of their sense of smell. However, as can be seen in the column labeled Classification, individuals’ perceptions of their olfactory abilities did not always coincide with the results obtained. This situation is not unusual as people are often inaccurate when rating their own abilities [48]. For instance, the subjects C04, C06, and C07 obtained a score corresponding to the hyposmia group despite believing they had a normal sense of smell, while C21, C23, C25, and C33 were in the opposite situation, obtaining a normosmic score.

Some participants had scores near the range limits, and their classification could be dubious. Since there is no pre-infection score available to compare their degree of recovery, classification based on score alone cannot indubitably categorize these patients’ sense of smell. Thus, the performance percentile according to their age group was also taken into consideration. Patients who obtained a performance percentile lower than 50% and agreed to continue with the study entered the stimulation phase.

### 3.2. EEG Data from Initial Olfactory Assessment

The purpose of analyzing the EEG data was to determine the brain regions that would be suitable for stimulation using the tDCS strategy. To achieve this, the topographic power levels during olfactory stimulation were studied, and differences between the participants who had and had not recovered their sense of smell were analyzed. The alpha band power produced in each trial after the presentation of the target stick was calculated for each subject and electrode, and the value was averaged between the trials answered correctly or incorrectly. The difference between the values of the correct and incorrect trials was then stored for each subject and electrode.

**Figure 3:**
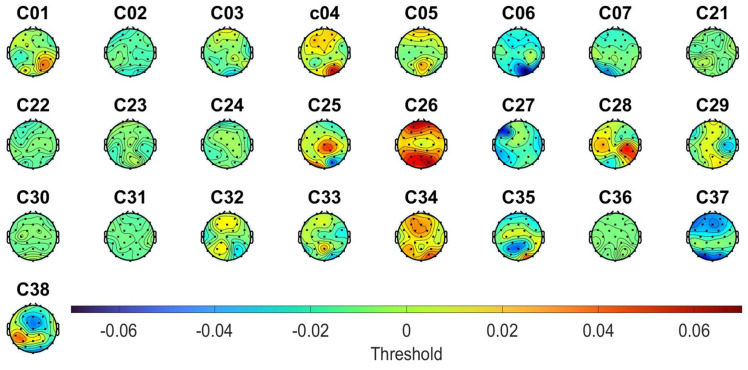
Differences between the alpha power when the participant correctly and incorrectly identified the target stick in the threshold test of the first olfactory assessment.

However, due to the heterogeneity of the data related to the power level during the olfactory stimulation (exemplified in Figure 3 for the case of the threshold test), it was not possible to identify any topographic pattern, even when considering all the subjects or dividing them based on their classification score. Thus, the selection of the electrode placement for the tDCS strategy was based solely on the information from the literature.

### 3.3. Stimulation

From the state of the art review, we verified that similar studies that employed tDCS in post-COVID-19 anosmia patients focused on the stimulation of the orbitofrontal cortex and studied olfactory improvements by analyzing only one olfactory ability (threshold in [30] and identification in [49,50]). However, the placement of the anode and cathode electrodes on the prefrontal cortex implies that the current is not going to affect the structures more profoundly, i.e., it will not reach all olfactory structures. This is shown in [30], where the stimulation is mainly of the trigeminal nerve, meaning that the olfactory improvements seen in this study could only be noticed for trigemic smells.

On the other hand, in [49], the participants received olfactory training during the stimulation, being used to some of the smells presented during the evaluation. The repetition of this presentation, 10 sessions of stimulation and 4 olfactory tests, may cause the patients to recognize these specific odors or memorize the options presented, which could compromise the correlation between the improvement of olfactory abilities and the evolution of the score. There is also the protocol proposed in [50], but there have not been published any results with this protocol yet.

Regarding the studies about anosmia caused by neurodegenerative diseases, they also focus on the secondary and tertiary olfactory structures, e.g., Ref. [24].

Our goal was to design a tDCS strategy to stimulate all the areas related to the olfactory system, not only the central olfactory system. Following this consideration, five models were contemplated.

1. Model 1: Anode electrodes, FP1 and FP2; cathode electrodes, P9 and P10.
2. Model 2: Anode electrodes, FP1 and FP2; cathode electrodes, O1, O2, and IZ.
3. Model 3: Anode electrodes, FPZ; cathode electrodes, IZ.
4. Model 4: Anode electrodes, FPZ and CZ; cathode electrodes, IZ.
5. Model 5: Anode electrodes, FPZ and CZ; cathode electrodes, P9 and P10.

The models were simulated; their corresponding normE values were retrieved and are presented in Table 2.

**Figure 4:**
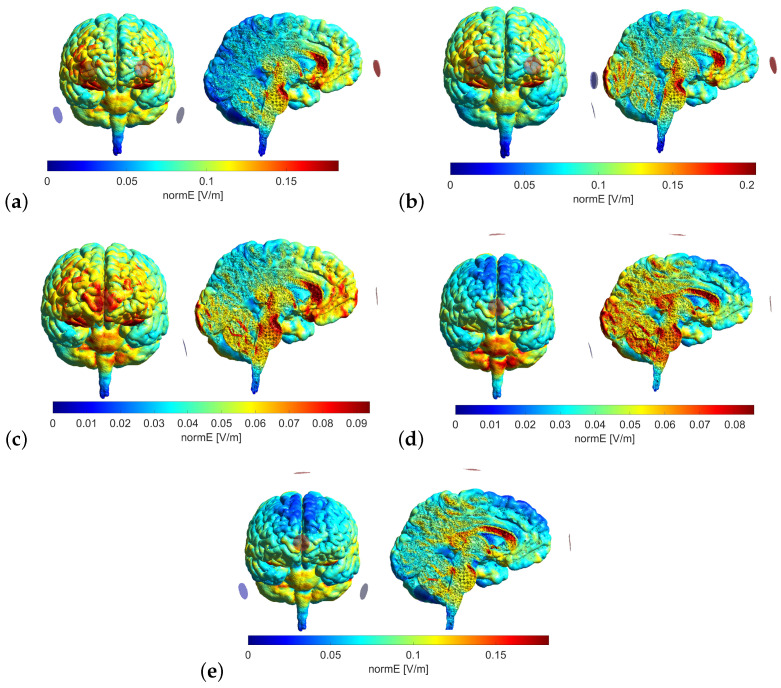
Representation of the different electrode configurations designed for the tDCS strategy, Models 1 to 5 represented in subfigures (**a**–**e**), respectively.

When comparing the areas stimulated in each model (see Figure 4) with the areas related to the olfactory system (such as the olfactory cortex, olfactory bulb, entorhinal cortex or orbitofrontal cortex), Models 3 and 4 are the ones that are more focused on the regions of interest. However, in Model 4, the presence of an anode electrode on position CZ produces a higher influence on the occipital cortex, while in Model 3, it is the frontal cortex that is more affected. Furthermore, the value of normE obtained in Model 4 is lower. Therefore, after considering all these factors, Model 3 was selected for the tDCS strategy.

### 3.4. Final Olfactory Assessment

Table 3 presents the scores obtained from the final olfactory test, which was conducted after the completion of the stimulation sessions, as well as a comparison with the initial values from the first evaluation.

To assess the normality assumption of each sample, the Shapiro–Wilk and Shapiro–Francia tests [51] were employed based on the kurtosis range of each sample. It was determined that all samples exhibited a normal distribution (*p* < 0.01). It is noteworthy to mention that the anosmic group could not be included in this study due to the limited number of available samples, which comprised only two cases.

A Kruskal–Wallis test was performed to examine potential subject dependency, and the results indicated no statistically significant differences among the subjects (*p* > 0.05). Additionally, a Mauchly’s test was performed to verify the assumption of sphericity, confirming the absence of any violations and thereby making any corrective measures unnecessary.

Subsequently, a two-way repeated measures ANOVA was conducted to compare the initial and final olfactory assessments across three distinct groups, henceforth referred to as stimulation groups: (1) patients classified as hyposmic who received active stimulation, (2) patients classified as anosmic who received active stimulation, and (3) patients classified as hyposmic who received sham stimulation. Each olfactory assessment score was individually analyzed using this statistical test.

The outcomes of this test are presented in Table 4. The obtained *p*-values solely permit the identification of statistically significant differences concerning the aforementioned groups, with all olfactory tests displaying *p*-values below 0.05.

In pairwise comparisons, it was observed that the differences in olfactory scores consistently had *p*-values greater than 0.998, except for the anosmic group in the discrimination test, where *p* = 0.71. This suggests that although a variation in mean values between the initial and final olfactory tests exists (as shown in Table 5), this difference lacks statistical significance.

Furthermore, the evolution of TDI scores in the anosmic group compared to the other groups is noteworthy. The anosmic group exhibited a statistically significant difference in the initial measurement compared to group 3 (*p* < 0.05) and group 1 (*p* < 0.1). However, this effect was not prominent in the last assessment (*p* > 0.1 in both cases). This suggests that the anosmic group exhibited a disparity in their TDI scores between assessments (with a mean initial TDI score of 15.38±0.18 and a mean final TDI score of 17.00±1.41), and although this variance lacks statistical significance, it results in the inability to further identify statistically significant differences in the hyposmic groups. A similar pattern was observed in the discrimination test, albeit less pronounced, whereas no such pattern emerged in the other two tests, where their final score was lower than their initial score.

Regarding the impact of each variable, the stimulation groups had the greatest effect. However, it was not remarkably substantial, as it did not exceed 0.20 in any individual test and only reached 0.35 in the TDI score.

Finally, a linear regression analysis was performed, with the final olfactory assessment scores as the dependent variable and the initial olfactory assessment scores or the time elapsed until the last olfactory test as independent variables. The patients were divided into those who received active or sham stimulation.

The patients who underwent sham stimulation did not seem to correlate their improvement with their initial score. The subject dependency seems to be the most influential variable. For instance, the subject with the highest initial TDI score, C23, obtained 3.75 points less in the retest, while the second subject with the highest initial TDI score, C21, exhibited an improvement of 5.5 points. The line of tendency of this group was y=0.1253x+27.679, with $R^{2}=0.0249$, where *y* is the initial TDI score and *x* is the difference between TDI scores. This equation would suggest that subjects with higher initial TDI scores would demonstrate greater improvement. However, it lacks reliability due to the low coefficient of determination, which indicates that the linear correlation between the variables does not explain the variances of the results obtained.

In contrast, the overall tendency of the subjects who received active stimulation might be that the initial score obtained influenced the amount of progression achieved, with greater improvement being observed among participants with lower scores. Specifically, those subjects who obtained anosmic scores in the first olfactory test showed an improvement in performance, while those with hyposmic values exhibited a decrease in performance (only one of them improved their TDI score, but by less than one point, which could also be attributed to test repetition).

The line of tendency for the active stimulation group was y=−1.1229x+23.501, with $R^{2}=0.5156$. As previously mentioned, this equation could explain approximately 50% of the cases by dividing the initial TDI scores lower than 23.5 into the group that is expected to show olfactory improvement, while the group with values higher than 23.5 would be expected to demonstrate lower final TDI scores.

The effect of the time elapsed between the last olfactory test and the last stimulation session or first olfactory test seems to be irrelevant since the distribution of improvement and worsening was similar for both groups of stimulation. Furthermore, all lines of tendency exhibit a low coefficient of determination. The $R^{2}$ values for the sham and active stimulation groups were 0.0208 and 0.0123, respectively, for the correlation between the time elapsed between the last olfactory test and the last stimulation session, and 0.1552 and 0.0186, respectively, for the correlation between the time elapsed between the last olfactory test and the first olfactory test. In the study described in [49], the results after a certain amount of time were similar, i.e., there was no clear improvement or worsening, although in their case, the time period was three months.

### 3.5. EEG Data from Final Olfactory Assessment

In order to assess the effects of brain stimulation, the differences between the first and last olfactory test for each trial were analyzed. The alpha band power produced in each trial after the presentation of the target stick was calculated for each subject and electrode using the same procedure as in the previous case. The trials of each test were then averaged into four groups: (1) trials that were answered correctly in both the first and last olfactory tests; (2) trials that were answered incorrectly in the first test, but correctly in the retest; (3) trials that were answered incorrectly in both tests; and (4) trials that were answered firstly correctly and later incorrectly.

**Figure 5:**
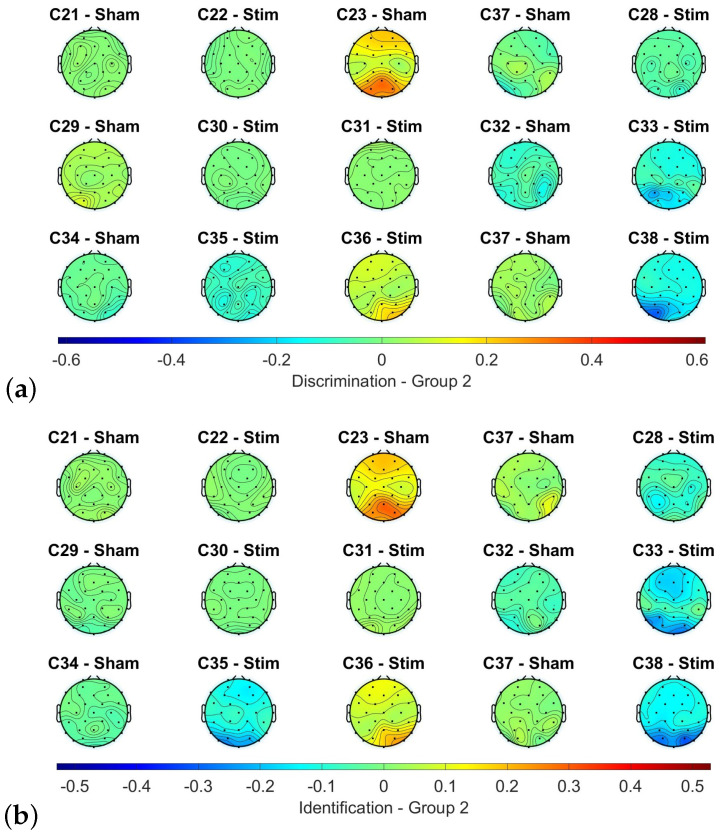
Differences between the alpha power between the first and last measurements of the trials first answered incorrectly and last correctly in the discrimination (**a**) and identification (**b**) tests.

Following five stimulation sessions, a comparison of the olfactory responses between the evaluations revealed different outcomes, with more than half of the subjects exhibiting similar responses in both tests. For instance, in group 2, which consisted of trials that were answered incorrectly in the initial test but correctly in the retest, most electrodes showed a difference close to zero for both the discrimination and identification tests. This is illustrated in Figure 5, where these electrodes are coloured green. However, some subjects showed higher or lower power levels during the final assessment. Specifically, subjects C23 and C36 exhibited higher power levels during the final assessment, particularly in the frontal and occipital area, while subjects C33, C35, and C38 showed higher power levels during the initial assessment, mainly in the occipital area. However, this variation does not seem to be associated with any of the analyzed variables, such as the type of stimulation received, the initial classification, or the level of improvement achieved. To illustrate this statement, some examples are presented. Subjects C33, C35, C36, and C38 all received active stimulation, but only C36 showed a higher power level. Additionally, both C23 and C33 had normosmic TDI scores, while C38 had an anosmic score. Lastly, subject C33 displayed a higher power level during the initial assessment for both tests, and while there was an improvement of one point in discrimination, the subject scored five points less in identification.

Despite the subject heterogeneity, the tendency between the initial and final assessments seems to have a degree regularity for each subject during the various tests, with subfigures (a) and (b) of Figure 5 exhibiting similarities with minor discrepancies.

**Figure 6:**
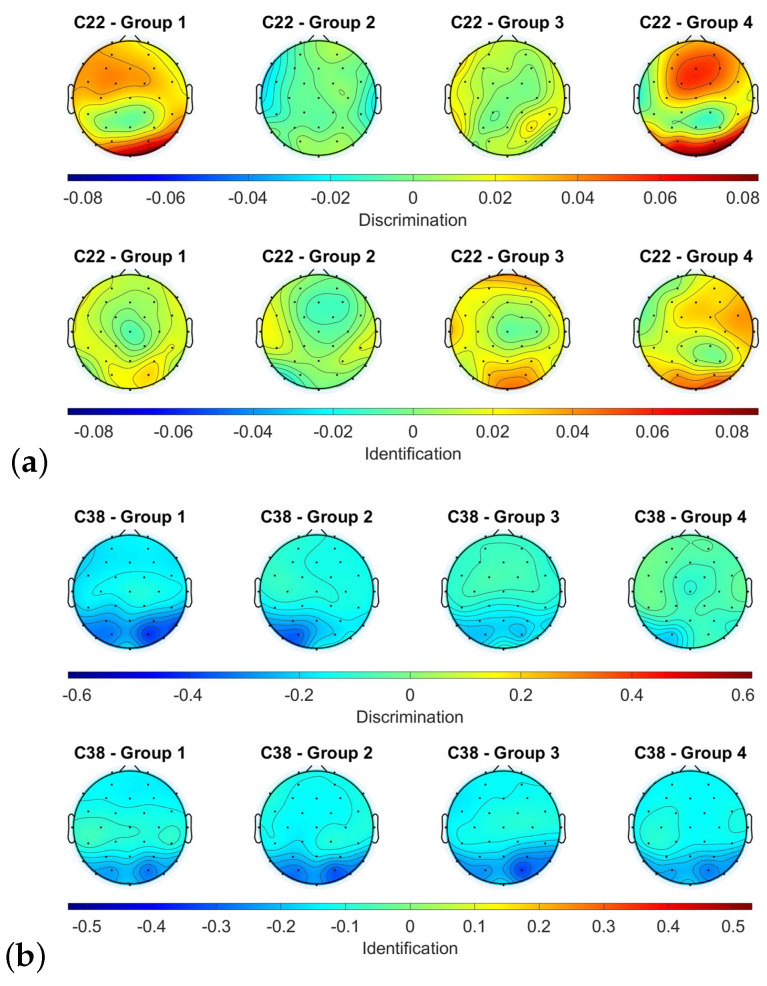
Differences between the alpha power between the different groups for the discrimination and identification tests of the subjects C22 (**a**) and C38 (**b**).

Regarding the different groups, the power level state variation is subject-dependent. For instance, subjects C22 and C38 (who both received active stimulation), as depicted in Figure 6, represent an example of heterogeneity in topological patterns. C22 exhibited a higher power level in the discrimination test of the last olfactory assessment in the fronto-parietal and occipital cortex for groups 1 and 4, whereas in groups 2 and 3, the subject responded similarly in both groups. In the identification test, groups 1 and 2 displayed similar responses, while groups 3 and 4 had higher power levels in the frontal and occipital cortex. On the other hand, C38 displayed similar responses for all groups and tests.

## 4. Discussion

### 4.1. Olfactory Assessments

In assessing the impact of neurostimulation on olfactory improvement, it was observed that the sham procedure had no influence regarding improving performance, which is consistent with the understanding that sham stimulation produces no effect on the brain. On the other hand, active stimulation was found to be effective only for individuals with anosmic scores. The group of participants with anosmia achieved a final TDI score of 17±1.41, with an initial TDI score of 15.38±0.18. In contrast, the hyposmic group under active stimulation obtained a lower final TDI score of 25.75±3.25 compared to their initial TDI score of 26.96±5.3. On the other hand, the group that received sham stimulation did not exhibit any change in their mean TDI score, with their final and initial TDI scores of being 27.68±6.79 and 27.68±3.93, respectively. Further details regarding the evolution of each individual test can be found in Table 5. However, it is important to note that the observed evolution of olfactory assessments across all studied groups did not reach statistical significance. The limited sample size of the study should be considered in evaluating the reliability of the obtained results.

It remains to be evaluated whether subjects who achieved hyposmic TDI scores would exhibit an improvement in their performance with modifications of the tDCS strategy, such as increasing the number of sessions or varying the electrode positions or current.

### 4.2. EEG Data

The analysis of EEG data presented a significant variety of results, making it challenging to draw conclusive findings. The analysis of EEG data is highly subject-dependent, and this study was no exception. Initially, a sample of 20 patients was allocated to stimulation, but this was later reduced to 15 after some patients declined to continue with the experimental procedure. The sample size, while not large, is noteworthy in comparison to similar studies and considering the one-year constraint for the project. The investigations described in [30,49,50] had 7, 10, and 20 subjects, respectively. However, the final groups were formed by only 3 or 4 patients, which makes it more difficult to identify any patterns. It would be interesting to increase the sample size in future developments. This would allow us to determine the possibility of different clusters within each group of analysis.

Furthermore, the analysis was limited by the data that were recorded. The Sniffin’ Sticks were useful in obtaining an objective and numerical value of the level of anosmia and its evolution, but the numerous variations in the properties of the sticks (different odors and different concentrations) complicated the association with the different EEG trials.

### 4.3. Stimulation

The diversity of values obtained in the analysis of the olfactory measurements and EEG data did not allow to identify in detail the effects that the stimulation caused. However, the fact that some subjects obtained scores in the last olfactory assessment over 46% give us confidence regarding the groundwork that this study can provide. Nonetheless, a study focused on improvements in the tDCS strategy is needed for those subjects that did not reach those goals.

The placebo effect that affected some subjects should also be mentioned. This can be seen in the case of patients C31 and C35, who achieved an improvement of almost 20%.

## 5. Conclusions

This paper has presented a potential approach based on tDCS to address anosmia post-COVID-19, a condition that currently lacks an effective treatment. The material employed enabled a comprehensive olfactory assessment based on three different abilities, as well as a precise stimulation of the targeted areas. The results obtained from the neurostimulation procedure have shown that subjects with lower scores in the olfactory assessment could experience an improvement in their condition, although this progression did not reach statistical significance. Therefore, it will be worthwhile in the future to perform more tests of this strategy with more patients and to even consider increasing the number of simulation sessions. The need for a larger sample size remains one of the primary limitations of this study. Additionally, it would be worthwhile to explore the application of this strategy in patients experiencing olfactory dysfunction due to other viral infections or neurodegenerative diseases, expanding the scope of its potential applications.

## Figures and Tables

**Table 1:** Results obtained in the first olfactory test.

Subject	Threshold	Discrimination	Identification	TDI	Classification	Percentile
C01	10.5	13	11	34.5	Normosmia	50–75%
C02	12.5	15	12	39.5	Normosmia	75–90%
C03	12.5	12	14	38.5	Normosmia	75%
C04	1	11	11	23	Hyposmia	0–5%
C05	11.75	12	13	36.75	Normosmia	50–75%
C06	6.25	13	14	33.25	Normosmia	25–50%
C07	6.75	12	8	26.75	Hyposmia	0–5%
C21	9.75	9	13	31.75	Normosmia	10–25%
C22	1.75	11	4	16.75	Hyposmia	0–5%
C23	11.25	12	10	33.25	Normosmia	25–50%
C24	5.5	11	8	24.5	Hyposmia	0–5%
C25	13.5	12	14	39.5	Normosmia	90%
C26	9	12	4	25	Hyposmia	0–5%
C27	4.5	9	12	25.5	Hyposmia	5–10%
C28	10.5	11	8	29.5	Hyposmia	5–10%
C29	12.5	9	7	28.5	Hyposmia	0–5%
C30	6.5	12	10	28.5	Hyposmia	10–25%
C31	7.5	11	7	25.5	Hyposmia	0–5%
C32	3.5	13	8	24.5	Hyposmia	0–5%
C33	9.25	13	9	31.25	Hyposmia	10–25%
C34	4.25	9	9	22.25	Hyposmia	0–5%
C35	9.25	11	10	30.25	Hyposmia	5–10%
C36	1.5	7	7	15.5	Anosmia	0–5%
C37	6	12	10	28	Hyposmia	0–5%
C38	3.25	4	7	14.25	Anosmia	0–5%

**Table 2:** Values calculated of normal component of the electric field for each model simulated.

	Point 1 [V/m]	Point 2 [V/m]	Point 3 [V/m]	Maximum [V/m]
Model 1	0.0982	0.1302	0.0848	0.1827
Model 2	0.0993	0.1324	0.0915	0.206
Model 3	0.0585	0.0704	0.0458	0.0939
Model 4	0.0387	0.0537	0.0389	0.0855
Model 5	0.0777	0.1043	0.0722	0.1821

**Table 3:** Evolution of the results of the olfactory assessment separated by the kind of stimulation received, being indicated by the initial and final score as well as the difference in points between each individual test (THR, threshold; DR, discrimination, and ID, identification) and combination score (TDI score). We have also indicated some dependent variables for each subject, such as their age, gender, days passed between the olfactory tests, and days between the last stimulation received and the final olfactory test.

**Stim Subject**	C22	C28	C30	C31	C33	C35	C36	C38
**Age**	52	40	54	29	46	34	28	42
**Gender**	M	F	M	M	F	M	F	F
**Time Olfactory Tests**	128	77	63	95	90	34	52	28
**Time Stimulation—Test**	6	14	4	1	6	1	3	14
**THR Initial**	1.75	10.5	6.5	7.5	9.25	9.25	1.5	3.25
**THR Final**	2.5	10.25	8.25	1	8	9.5	1	1
**THR Difference**	0.75	−0.25	1.75	−6.5	−1.25	0.25	−0.5	−2.25
**DR Initial**	11	11	12	11	13	11	7	4
**DR Final**	12	6	13	13	14	9	9	9
**DR Difference**	1	−5	1	2	1	−2	2	5
**ID Initial**	4	8	10	7	9	10	7	7
**ID Final**	10	8	8	7	4	11	8	6
**ID Difference**	6	0	−2	0	−5	1	1	−1
**TDI Initial**	16.75	29.5	28.5	25.5	31.25	30.25	15.5	14.25
**TDI Final**	24.5	24.25	29.25	21	26	29.5	18	16
**TDI Difference**	7.75	−5.25	0.75	−4.5	−5.25	−0.75	2.5	2.25
**Sham Subject**	C21	C23	C27	C29	C32	C34	C37	
**Age**	23	25	53	37	53	55	30	
**Gender**	F	M	F	M	M	F	M	
**Time Olfactory Tests**	116	89	132	82	62	91	23	
**Time Stimulation—Test**	7	4	8	11	6	7	6	
**THR Initial**	9.75	11.25	4.5	12.5	3.5	4.25	6	
**THR Final**	10.25	10.5	1	10.25	3	6.75	10	
**THR Difference**	0.5	−0.75	−3.5	−2.25	−0.5	2.5	4	
**DR Initial**	9	12	9	9	13	9	12	
**DR Final**	15	6	6	12	11	10	13	
**DR Difference**	6	−6	−3	3	−2	1	1	
**ID Initial**	13	10	12	7	8	9	10	
**ID Final**	12	13	11	8	7	8	10	
**ID Difference**	−1	3	−1	1	−1	−1	0	
**TDI Initial**	31.75	33.25	25.5	28.5	24.5	22.25	28	
**TDI Final**	37.25	29.5	18	30.25	21	24.75	33	
**TDI Difference**	5.5	−3.75	−7.5	1.75	−3.5	2.5	5	

**Table 4:** ANOVA analysis displaying the sum of squares, degrees of freedom, mean square, F value, *p*-value, and omega squared. This test evaluated the effect of the stimulation groups and the repetition of the olfactory assessment, as well as the combination of both variables for each individual test and for the TDI score.

THR						
	**Sum of Squares**	**df**	**Mean Squares**	**F**	***p*-value**	$\omega^{2}$
**Group stimulation**	107.05	2	53.52	4.27	0.0258	0.18
**Olfactory assessment**	3.13	1	3.13	0.25	0.6218	−0.03
**Group stimulation × Olfactory assessment**	2.05	2	1.03	0.08	0.9215	−0.07
**Residual**	300.51	24	12.52			
DR						
	**Sum of Squares**	**df**	**Mean Squares**	**F**	**p-value**	** $\omega^{2}$ **
**Group stimulation**	50.12	2	25.06	4.17	0.0279	0.18
**Olfactory assessment**	6.19	1	6.19	1.03	0.3202	0.001
**Group stimulation × Olfactory assessment**	11.75	2	5.88	0.98	0.3908	−0.002
**Residual**	144.26	24	6.01			
ID						
	**Sum of Squares**	**df**	**Mean Squares**	**F**	**p-value**	** $\omega^{2}$ **
**Group stimulation**	32.9	2	16.45	3.4	0.0502	0.14
**Olfactory assessment**	0.15	1	0.15	0.03	0.8598	−0.03
**Group stimulation × Olfactory assessment**	0.22	2	0.11	0.02	0.9779	−0.07
**Residual**	116.21	24	4.84			
TDI						
	**Sum of Squares**	**df**	**Mean Squares**	**F**	**p-value**	** $\omega^{2}$ **
**Group stimulation**	421.68	2	210.84	8.9	0.0013	0.35
**Olfactory assessment**	0.11	1	0.11	0	0.9469	−0.04
**Group stimulation × Olfactory assessment**	6.49	2	3.24	0.14	0.8727	−0.06
**Residual**	568.64	24	23.69			

**Table 5:** Descriptive statistics (mean ± standard deviation) of different groups of patients formed according to the type of stimulation received, the classification regarding their initial TDI score, or a combination of both variables calculated for each individual test and TDI score for the initial and final olfactory assessment.

	Stim—Anosmia	Stim—Hyposmia	Stim	Sham—Anosmia	Sham—Hyposmia	Sham
**THR Initial**	2.38±1.24	7.46±3.14	6.19±3.58	-	7.39±3.69	7.39±3.69
**DR Initial**	5.5±2.12	11.5±0.84	10±2.98	-	10.43±1.81	10.43±1.81
**ID Initial**	7.5±0.71	8±2.28	7.88±1.96	-	9.86±2.12	9.86±2.12
**TDI Initial**	15.38±0.18	26.96±5.37	24.06±7.03	-	27.68±3.93	27.68±3.93
**THR Final**	1±0	6.58±3.86	5.19±4.16	-	7.39±3.94	7.39±3.94
**DR Final**	9±0	11.17±3.06	10.63±2.77	-	10.43±3.41	10.43±3.41
**ID Final**	7±1.41	8±2.45	7.75±2.19	-	9.86±2.27	9.86±2.27
**TDI Final**	17±1.41	25.75±3.25	23.56±4.92	-	27.68±6.79	27.68±6.79
	**Anosmia**	**Hyposmia**	**Total**			
**THR Initial**	2.38±1.24	7.42±3.3	6.75±3.55			
**DR Initial**	5.5±2.12	10.92±1.5	10.2±2.43			
**ID Initial**	7.5±0.71	9±2.31	8.8±2.21			
**TDI Initial**	15.38±0.18	27.35±4.46	25.75±5.9			
**THR Final**	1±0	7.02±3.76	6.22±4.08			
**DR Final**	9±0	10.77±3.14	10.53±2.97			
**ID Final**	7±1.41	9±2.45	8.73±2.4			
**TDI Final**	17±1.41	26.79±5.33	25.48±6.03			

## Data Availability

Not applicable.

## Sample Availability

The datasets presented in this article are not readily available because of the Ethics Committee for Drug Research of the Department of Health of Alicante—General Hospital review board. Requests to access the datasets should be directed to Dr. Azorín.
